# Influence of Cement Kiln Dust on Long-Term Mechanical Behavior and Microstructure of High-Performance Concrete

**DOI:** 10.3390/ma17040833

**Published:** 2024-02-09

**Authors:** Piotr Smarzewski, Krystian Błaszczyk

**Affiliations:** 1 Faculty of Civil Engineering and Geodesy, Military University of Technology, 2 Gen. Sylwestra Kaliskiego, 00-908 Warsaw, Poland; 2 Candidate for Doctoral School, Military University of Technology, 2 Gen. Sylwestra Kaliskiego, 00-908 Warsaw, Poland

**Keywords:** high-performance concrete, cement kiln dust, long-term behavior, compressive strength, elastic modulus, splitting tensile strength, bulk density, porosity, water absorption, ultrasonic pulse velocity, microstructure

## Abstract

Cement production in the world market is steadily increasing. In 2000, it was 1600 million tons, while as of 2013, the annual amount exceeded 4000 million tons. The burning of cement clinker is associated with the generation of waste. It is estimated that the amount of cement kiln dust (CKD), during combustion, reaches about 15–20%, which means 700 million tons per year. However, not all types of by-products are reusable due to high alkali, sulfate, and chloride contents, which can adversely affect the environment. One environmentally friendly solution may be to use CKD in the production of high-performance concrete (HPC), as a substitute for some of the cement. This paper presents a study of the short- and long-term physical and mechanical properties of HPC with 5%, 10%, 15%, and 20% CKD additives. The experiments determined density, water absorption, porosity, splitting tensile strength, compressive strength, modulus of elasticity, ultrasonic pulse velocity, and evaluated the microstructure of the concrete. The addition of CKD up to 10% caused an increase in the 28- and 730-day compressive strengths, while the values decreased slightly when CKD concentration increased to 20%. Splitting tensile strength decreased proportionally with 5–20% amounts of CKD regardless of HPC age. Porosity, absorbability, and ultrasonic pulse velocity decreased with increasing cement dust, while the bulk density increased for HPC with CKD. Microstructure analyses showed a decrease in the content of calcium silicate hydrate (C–S–H), acceleration of setting, and formation of wider microcracks with an increase in CKD. From the results, it was shown that a 15% percentage addition of CKD can effectively replace cement in the production of HPC and contribute to reducing the amount of by-product from the burning of cement clinker.

## 1. Introduction

Cement kiln dust (CKD) is a fine-grained, highly alkaline material removed from exhaust gases by means of air pollution control tools. Fabric dust collectors or electrostatic precipitators are used for this purpose. Dust is generated in the production of cement in a rotary kiln during the burning of the clinker. Due to the significant increase in the by-product CKD, ways are being found to use it.

The chemical composition of CKD depends on both the raw materials used to produce clinker and the source and type of coal fuel used to heat the material in the rotary kiln. Free lime can be found in CKD and its concentration is usually highest in the coarser particles collected closest to the furnace. Finer particles tend to exhibit higher concentrations of sulfates and alkalis. Coarser particles that have not been separated and returned to the kiln mean that the dust will contain a greater amount of free lime [1].

Hefni et al. [2] described a situation in which significant amounts of CKD, due to excessive concentrations of alkali, chloride, and sulfate, are not subjected to utilization processes and are landfilled. CKD has potential for use in agriculture; the addition of dust can provide plants with elements such as sulfur, magnesium, and potassium, thereby replacing chemical fertilizers [3]. Due to the favorable amounts of micro and macronutrients, CKD is an efficient source of calcium and potassium, which are essential for potato growth, comparable to fertilizers, while not contaminating soils with heavy metals [4]. Dinel et al. [5] examined the chemical distribution of trace metals (Cu, Zn, Cr, Pb, Ni, Co, and As) in soil after a single application of CKD and its effect on soybean growth. The total concentration of metals in the biomaterials was relatively low compared to the limits set by the US Environmental Protection Agency.

Mackie and Walsh [6] demonstrated the removal efficiency of total suspended solids by CKD. They compared the efficiency of purification of Ca(OH)_2_ suspended solids by using four different CKD samples with a control treatment of CaO quicklime. All samples allowed for the precipitation of more than 99% of soluble zinc and iron. In addition, sedimentation experiments showed that all CKD samples produced less sediment by volume than samples to which quicklime was added.

El-Awady et al. [7] analyzed the possibility of cleaning sludge using CKD at a high pH value. It was optimal to add CKD at 8 g/L to the primary sludge and 7 g/L to the return sludge. The removal efficiencies obtained for BOD5, COD, and PO4 were 99.2, 99.5, and 93.9%, respectively. These results were confirmed by Mostafa [8], who found that the addition of 2 g/L allowed the removal of BOD5, COD, PO4, and phosphate at 94.1, 94.1, 94.2, and 87.9%, respectively. In addition, swelling was found to be reduced in the temperature range of 15–20 °C. Taha et al. [9] presented the results of a study in which CKD was employed as an ion adsorbent, varying the length of contact time and pH of the solution. The addition of CKD allowed the removal of heavy metals such as Zn (80–99%), Co (50–90%), Cd (90–99%), and Al (85–99%) from wastewater, at pH ranging from 5.5 to 8.

Another use of CKD dust is soil stabilization. The effect of applying CKD on the properties of a ternary road binder was analyzed [10]. For road applications, the possibility of the proportion of CKD up to a maximum of 40% was obtained. Peethamparan et al. [11] reported a positive effect of CKD on the compressive strength of samples compacted with four different dust samples. In addition, they reported that a slight increase in the hydration temperature with the addition of 25% CKD (1–8 °C), did not reduce the safety of carrying out soil stabilization work. Adaska and Taubert [12] proved that it was effective for expansive soils due to its similarity to cement, calcium, and fly ash. The use of CKD in the stabilization of clay soils showed an increase in strength and an improvement in the plasticity index. In addition, the soil’s frost resistance was increased, and CKD was proven to have applications in solidification and waste stabilization, including hazardous substances. 

CKD can be used to neutralize aqueous wastes, spent acids, caustics, fuel oils, salt solutions, metallic solids, crude oil, adhesives and epoxies, combustion residues, contaminated soils, organic paints, inks and varnishes, among others. CKD dusts, due to their high concentration of Ca and K, can be used to fertilize soils or be applied as fertilizer. They increase the effectiveness of herbicides and neutralize soil acidification. In the US, a soil additive consisting of 35% CaO, 6% MgO, 5% K_2_O, and 4% SO_3_ was developed from CKD dust. Australian researchers have proven that CKD in a granular form can significantly increase crop yields.

Another use of CKD is as an additive in mixtures for the production of building materials. Ghorab et al. [13], Aydin et al. [14], and Ahmari and Zhang [15] demonstrated the possibility of replacing some of the ingredients with CKD to reduce the cost of ceramic materials. El-Attar et al. [16] studied the effect of 30 and 50% cement replacement on CKD. They proved a decrease in the compressive strength of concrete after 28 days of maturation with an increase of 26% and 32.1% for 30% and 50% CKD, respectively, and an increase in water demand of 3.6% and 12.7%, respectively. Total carbon dioxide emissions decreased sharply (23% and 36%) with the increase in dust from the cement kiln. Standard technical specifications allow the manufacture of bricks for low cement content (75 kg/m^3^ CKD at 50% cement replacement). This resulted in a cost savings of up to 30%, thereby lowering the cost of building construction, and had a positive impact on environmental protection. Abd El-Hameed et al. [17] analyzed the impact of replacing part of the cement with CKD at 10, 20, 30, and 40%. They demonstrated the feasibility of using CKD in the production of concrete at an optimal concentration of 20%. Larger percentage additions of cement dust were considered unfavorable due to their negative effects on the properties of the concrete mixtures tested.

It is clear from the above literature review that there are still many problems to be overcome in the design and application of concrete with CKD additives. It is particularly interesting to determine the effect of partial replacement of cement by CKD on the long-term physical-mechanical characteristics of high-performance concrete (HPC). Previous studies of the effect of CKD additive were based on the analysis of properties after 7 to a maximum of 90 days of maturation and concerned low/normal strength concrete. Therefore, a comprehensive study of HPC mixtures with CKD additive is presented here, with the determination of parameters after two years of curing. The analysis of the HPC behavior over a long period of time minimizes the uncertainty associated with changes in the HPC properties and the additional data make it possible to determine the optimal addition of CKD to HPC.

Herein, the effect of CKD addition at 5, 10, 15, and 20% as a replacement for cement is investigated. Analyses were made for HPC with a constant water–cement ratio of w/c = 0.25. Compressive strength and splitting tensile strength were determined for individual concrete mixtures after 7, 28, and 730 days of maturation. In addition, the density, porosity, water absorption, modulus of elasticity, and microstructure of the samples were analyzed after 7 and 28 days of maturation, as well as the ultrasonic pulse velocity (UPV) after 730 days of curing.

## 2. Materials and Methods

### 2.1. Materials

CEM I 42.5 R cement (CEMEX, Chełm, Poland) meeting standards [18,19] was used in the production of HPC mixtures. Table 1 shows the basic recipe (without the addition of CKD) for 1 m^3^ of concrete mix. Sand of grain size 0.05–2 mm and granite aggregate of 2–16 mm was used. Aggregate gradation was determined according to the standard [20]. The water-to-binder ratio (W/B) for the tested HPCs was 0.25. Maintaining a low ratio was determined by the need to reduce alkaline reactions, which have an adverse effect on the strength of concrete. In order to achieve an appropriate level of workability of the mix, the superplasticizer CX ISOFLOW 755 (CEMEX Admixtures GmbH, Salzkotten, Germany) was used, with a light yellow color, density of 1.07 ± 0.02 g/cm^3^, pH 6.0 ± 1.0, with Cl^−^ ≤ 0.1% and Na_2_O ≤ 1.5%. The amount of superplasticizer in each subsequent mixture was higher by 2.5 L/m^3^ than the previous one, along with the increase in the percentage of CKD addition due to the changing water demand of the mixture.

As indicated in [21,22,23,24], the addition of CKD results in a higher demand for batch water. This can be attributed to the high content of alkalis, sulfates, and volatile salts. In addition, CKD contains very fine particles, which increases the surface area that requires binding. The average particle size of CKD is approximately 0.5 µm, which is much smaller than that of cement in which most particles are in the range of 3–30 µm.

Replacement of cement with CKD (CEMEX, Chełm, Poland) at 1.8 kg for 5%, 3.6 kg for 10%, 5.3 kg for 15%, and 7.1 kg for 20%. The chemical composition of CKD depends on factors at the cement plant, but is similar to typical Portland cement. The chemical composition of the CKD used is shown in Table 2.

**Figure 1:**
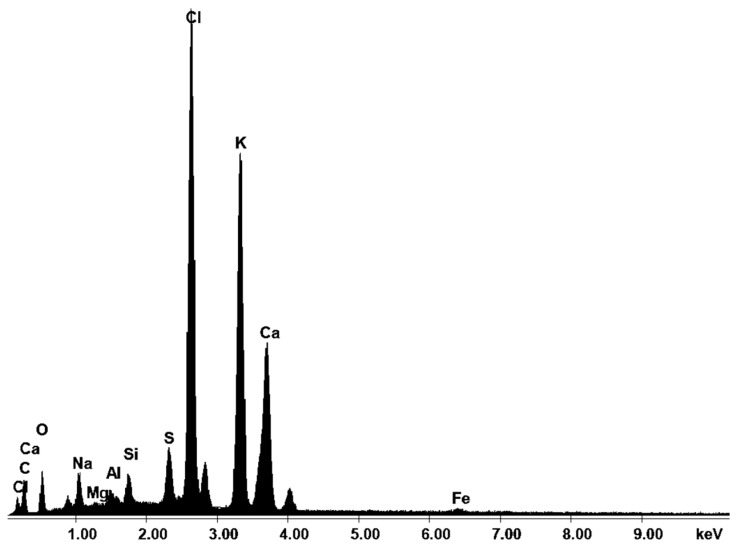
EDS spectrum of CKD.

The main components of CKD are compounds of calcium, potassium, iron, silica, and aluminum oxides. CKD has a higher concentration of alkali and sulfur compounds compared to Portland cement, which is the reason for restrictions on its reuse in cement or concrete production. CKD can also contain trace amounts of metals such as cadmium, lead, and selenium. Figure 1 shows the EDS spectrum of CKD used in HPC production.

From the above test, the elemental composition of the dust was determined to be comprised of 13.61% C (the CKD sample was sputtered with coal), 10.31% O, 2.60% Na, 0.31% Mg, 0.83% Al, 1.51% Si, 2.50% S, 26.90% Cl, 25.82% K, 14.78% Ca, and 0.82% Fe.

**Figure 2:**
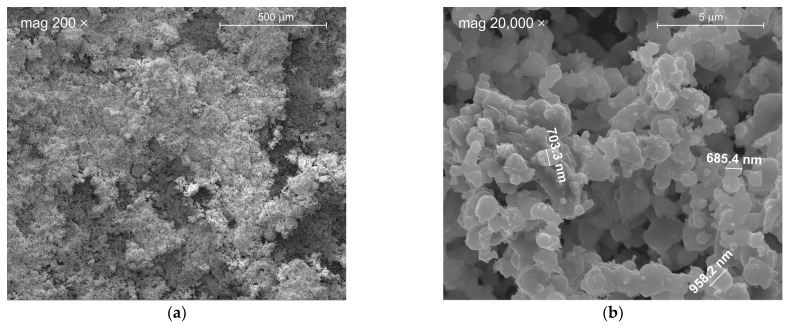
CKD under magnification (**a**) 200×; (**b**) 20,000×.

The low content of heavy metals, according to [12], does not affect the applicability of CKD. Tests were conducted under RCRA (Resource Conservation and Recovery Act) for the presence of eight metals: As, Ba, Cd, Cr, Pb, Hg, Se, Ag. Samples were additionally analyzed for antimony, beryllium, thallium, and nickel. The average level of metals found in CKD was well below the regulatory limits. During the chemical composition study, microphotographs of the kiln dust were taken at 200× magnification and 20,000×. Figure 2b shows the structure of the CKD with the dimensions of each dust particle marked.

Cubic specimens measuring 100 mm × 100 mm × 100 mm and cylindrical specimens measuring 150 mm in diameter × 300 mm in height were prepared for the tests. Five formulations of HPC mixtures were prepared (a control without dust addition and with 5, 10, 15, and 20% CKD addition). For each formulation, six cylindrical specimens and 18 cubic specimens were made. Compressive and tensile strengths were tested on the cubic specimens, while the modulus of elasticity was tested on the cylindrical specimens. The making of the mixture began with the preparation of concrete molds, then all the ingredients were combined in a concrete mixer. After mixing to obtain a homogeneous mass, the material was used to fill the molds and the vibration process was carried out. The specimens were allowed to stand for 24 h until they reached a strength that would allow them to be unmolded, and then they were placed in a tank filled with water for 14 days. After this time, the samples were kept in air-dry conditions until testing.

### 2.2. Absorbability Test

The concrete absorbability test was carried out in accordance with the standard [25]. Cubic specimens of 100 mm × 100 mm × 100 mm were used, one for each HPC. The tests were carried out after 28 days of maturation of the samples. The cubes were placed in a vessel on pads 10 mm thick. The vessel was then filled with water at 18 °C to half the height of the samples. After 24 h, the water level was raised to 10 mm above the samples and held until the cubes were completely saturated. The samples were weighed and allowed to absorb the water until subsequent weightings showed no change in weight. The final result was recorded. The samples were then placed in a drying oven at 105 °C until a constant weight was obtained and final weights were recorded. Water absorption by concrete (*A_w_*) in % to the nearest 0.01% was calculated according to the formula

(1) $$A_{w}=\frac{m_{s}-m_{d}}{m_{d}}\cdot 100\;(\%)$$

where: *m_d_*—dry sample weight (g), *m_s_*—mass of saturated sample (g).

### 2.3. Bulk Density and Porosity Tests

Testing of the bulk density of HPC was carried out in accordance with recommendations [26]. Cubic specimens of 100 mm × 100 mm × 100 mm, one of each mixture, were used for the test. The results were analyzed after 28 days of maturation, considering the samples maximally saturated with water and after surface drying with paper. A water density of 1 g/cm^3^ was assumed in the study. Open porosity was determined based on the following formula

(2) $$P_{o}=\frac{m_{s}-m_{d}}{m_{s}-m_{h}}\cdot 100\;(\%)$$

where: *P_o_*—open porosity (%), *m_d_*—dry sample weight (g), *m_s_*—mass of saturated sample (in air) (g), *m_h_*—mass of sample immersed in water (g).

### 2.4. Compressive Strength Test

The parameter describing the maximum compressive load carrying capacity is called compressive strength. The test was conducted on 100 mm × 100 mm × 100 mm specimens after 7, 28, and 730 days according to [27]. Acceptance of the test results depended on the correct failure pattern of the specimen. To determine the compressive strength, three specimens were used for each mix.

### 2.5. Splitting Tensile Strength Test

The test was conducted in accordance with the standard [28]. Cubic specimens of 100 mm × 100 mm × 100 mm were used and tested at 7, 28, and 730 days after forming. The load was applied centrally after the 4 mm wide fiberboard shims were spread over the length of the specimen. Three specimens for each HPC were used to determine the splitting tensile strength.

### 2.6. Modulus of Elasticity Test

The test was carried out on cylindrical specimens of 150 mm diameter and 300 mm height after 7 and 28 days of curing. To determine the elastic modulus, three specimens were used for each mixture. Before determining the modulus of elasticity, the upper and lower surfaces were sanded so that the stresses in the specimen during loading would propagate uniformly. The test was carried out in a hydraulic press by loading the rollers according to the direction of concreting.

### 2.7. Ultrasonic Pulse Velocity Test

The velocity of passage of the ultrasonic pulse between the transmitter and receiver enables assessment of the quality of the concrete. Usually, longitudinal waves with frequencies of 20 to 150 kHz are used. Transmitters should be placed on opposite sides of the specimen. The higher the homogeneity of the concrete, the less time is required for the wave to pass between the devices. The lowest velocities will occur in concrete with voids or many cracks. The test also allows the assessment of the type and depth of defects and the quality of repairs made [29].

### 2.8. Microstructure Analysis

The microstructure is the number, size, type, and distribution of the various phases of concrete. There are three basic phases of HPC: aggregate, hydrated cement slurry, and interfacial transition zone (ITZ). The local microstructure of HPCs was analyzed for differences depending on the amount of CKD added, using a scanning electron microscope (SEM). To this end, microphotographs of the samples were taken at magnifications ranging from 100 to 20,000 times and EDX analyses were performed to determine the chemical composition of the HPCs. Analyses were carried out after 28 days of maturation of the samples.

## 3. Results and Discussion

### 3.1. Absorbability

**Figure 3:**
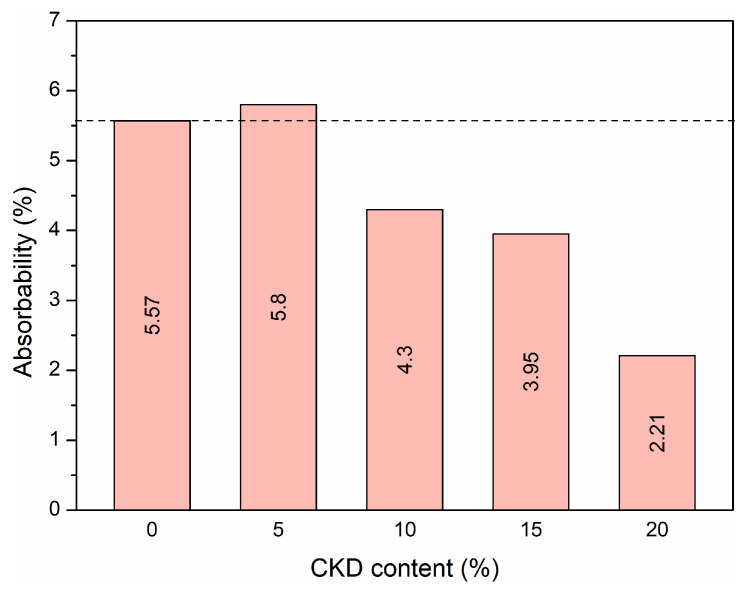
Absorbability after 28 days of maturation.

By comparing the weights of the dry and water-saturated samples, the absorbability of the concrete after 28 days of curing was calculated. The results are revealed in Figure 3.

HPC samples with 0 and 5% CKD addition achieved the highest absorbability, equal to 5.57% and 5.80%, respectively. As the concentration of dust increased further, the absorbability began to decrease, reaching a minimum of 2.21% for 20% CKD addition. The greater than two-fold decrease in water absorption means that less water can be found in the free spaces of the concrete, improving the properties that are particularly important when the concrete is exposed to direct contact with weather conditions. The lower water absorption in concretes with CKD addition due to phenomenon of dust particles filling the voids due to their high degree of fineness, as illustrated in Figure 2b.

Ahmed et al. [30] examined the absorbability of concrete for the addition of CKD at 5, 10, 15, and 20% after 28 days of curing. For this purpose, they made cylindrical samples with a diameter of 100 mm and a height of 200 mm. The highest saturation occurred for the control mix (0% CKD)—14.78%. As the amount of added dust increased, this value decreased to 13.14%, 13.07%, 12.26%, and 13.11%, for 5, 10, 15, and 20% CKD, respectively. They observed that the water absorption of the CKD samples decreased due to the high degree of particle refinement, which allows the voids to be filled. It was found that for 20% CKD the void ratio decreased by 2% and the voids were filled by dust particles with an average size of 0.5 µm, which reduced the possibility of water absorption.

Kadhim et al. [31] made concrete samples with the addition of CKD nanoparticles in amounts of 1, 2, 3, 4, and 5% in relation to the total weight of the mixture. For concrete after 7, 28, and 90 days of maturation, the saturation decreased with increasing dust content and the age of the samples. After 28 days, the results obtained were 3.40, 3.30, 3.27, 2.80, 2.40, and 2.26% and after 90 days, 1.95, 1.80, 1.65, 1.10, and 0.80%. In the case of concrete after 28 days of curing, the addition of 5% CKD caused a decrease in water absorption by 33%, while for the sample after 90 days, absorption decreased by 59%. The results indicate that the addition of CKD affects the water absorption in the long term.

It can be seen that water absorption decreased by a maximum of 60% with the addition of CKD to the mixtures. Therefore, CKD improved the absorption properties of concrete and thus can increase its durability. Concrete mixtures containing CKD always showed lower absorption values than the reference mixture without CKD. This can be attributed to the very fine size of the CKD particles.

### 3.2. Bulk Density and Open Porosity

**Figure 4:**
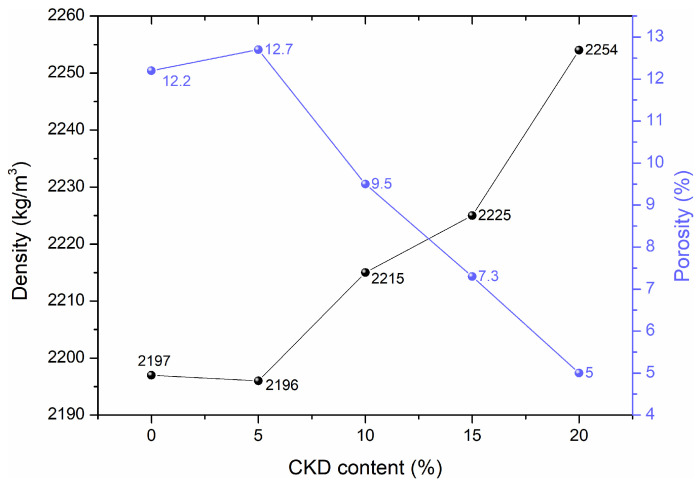
Bulk density and open porosity of HPCs after 28 days of maturation.

HPC density was shown to increase slightly with increasing amounts of CKD. The lowest density was achieved by 0 and 5% CKD samples, 2197 and 2196 kg/m^3^, respectively. The highest density of 2254 kg/m^3^ was achieved by a sample with 20% CKD addition. Comparing the density of 20% CKD to the control sample showed an increase of 2.59%. The increase in the density of the concrete samples was caused by small dust particles from the cement kiln that filled the voids that were empty in the reference mixture. The density of CKD is 2.6–2.8 g/cm^3^ compared to 1 g/cm^3^ of water and 0.0012 g/cm^3^ of air. The change in density also depends on differences in the size of dust particles from the cement kiln and cement, which is related to the degree of fineness. In the case of HPC, the individual ingredients are selected in an appropriate way, ensuring the ability to bind them without leaving a large number of unbound particles. The added CKD fills the free spaces, which increases the density. The bulk density results are presented in Figure 4.

The effect of the addition of CKD on the density of self-compacting concrete was presented in Kadhim et al. [31]. The addition of CKD in the amount of 1–5% influenced the density of samples after curing for 7, 28, and 90 days. The control sample after 28 days reached 2010 kg/m^3^, when CKD was added this value increased accordingly with the increased dust content, up to a maximum of 2040 kg/m^3^ for 5%. The results clearly indicate an increase in the density of self-compacting concrete with the addition of CKD. The high degree of fineness of CKD particles fills the voids due to the good workability of the mixture and proper mixing of the ingredients. Najim et al. [32] studied the chemical and physical properties of concrete with the addition of 10, 20, and 30% CKD. They obtained concrete densities of 2211 kg/m^3^, 2191 kg/m^3^, 2168 kg/m^3^, and 2105 kg/m^3^ for 0, 10, 20, and 30% CKD, respectively. In addition, a slight increase in the porosity was shown using 2D image analysis obtaining 3.26, 4.41, 6.23, and 7.25% with an increase in dust content up to 30% CKD. In the case of ordinary concrete, a decrease in density was achieved, unlike HPC and self-compacting concrete. Replacing cement with dust resulted in poorer bonding of the concrete mixture particles and the formation of voids. In the case of replacing the cement with 30% CKD, the density decrease was as much as 5%. This resulted in a significant change in the concrete matrix.

In our paper, open porosity values were calculated based on mass measurements. The control sample achieved a value of 12.2%. The mixtures with added dust achieved 12.7%, 9.5%, 7.3%, and 5% for 5, 10, 15, and 20% CKD, respectively. A gradual decrease in the open porosity was found with the addition of CKD. A dust concentration 20% decreased this parameter by almost 2.5 times. The results indicate that the addition of CKD significantly reduces the porosity of concrete. This is caused by filling the free voids with fragmented CKD particles.

Kadhim et al. [31] calculated the total porosity of concrete samples with the addition of CKD. For concrete after 7, 28, and 90 days of maturation, the porosity decreased with the increase in dust content and the age of the samples. The control sample (0% CKD) achieved 1.9% and 1.55% for 28 and 90 days, respectively. The addition of 5% CKD reduced the porosity to 1.25% and 0.75% after 28 and 90 days, respectively.

Kunal et al. [33] examined the properties of concrete with the addition of 5, 10, and 15% CKD after 7, 28, and 91 days of curing. It has been shown that porosity decreases as the amount of dust increases. In the case of concrete, no noticeable difference was observed after 7 days of curing. In each case, the addition of CKD caused a decrease in porosity. The lowest result was achieved by concrete with the addition of 10% dust after 28 days of maturation, decreasing by 41.3%. A further increase in concentration of CKD to 15% resulted in an increase in porosity. This was due to the decrease in cement content, which reduced the bonding of CKD in concrete and caused the formation of pores.

In contrast, in [34], the addition of CKD in amounts of 5, 10, 15, and 20% for normal strength concrete did not have a significant effect on the porosity of concrete. For all samples after 7 days of curing, the obtained values were in the range of 18.2–18.5%. After 28 days, with the addition of CKD, the porosity decreased slightly, reaching a maximum 17.2% for 20% dust. After 90 days of curing, the control sample without the addition of CKD reached 13.7% porosity and the sample with 20% CKD—14.0%. The porosity results for normal strength concrete show the same trend as density. The change in porosity depends on the type of concrete, its properties, and the differences between the grain size of cement and CKD. For ordinary concrete, the addition of CKD does not significantly affect the porosity results.

### 3.3. Compressive Strength

**Figure 5:**
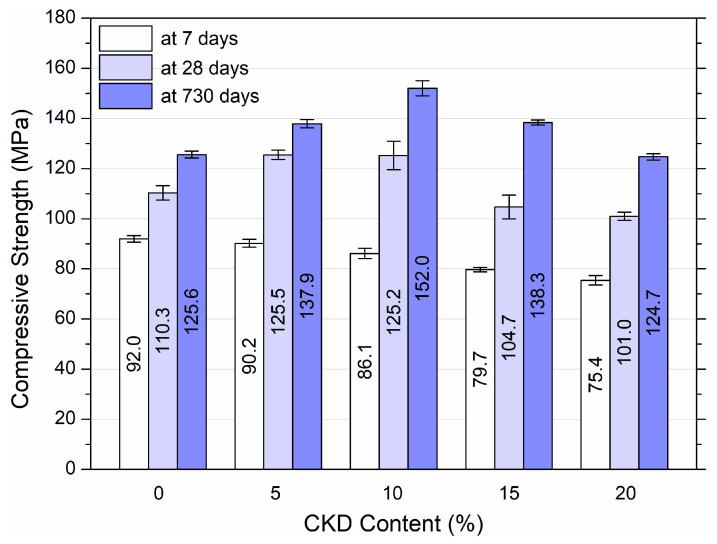
Compressive strength of HPC at 7, 28, and 730 days of maturation with variable addition of CKD.

Compressive strength tests were carried out using HPC samples. The results after 7, 28, and 730 days of maturation are shown in Figure 5.

The results illustrated in Figure 5 display the average compressive strength values with standard deviation for the three cube samples for each mixture. Based on the results, an influence of the concentration of CKD on the compressive strength of HPC was found. The strength after 7 days of maturation decreased with the increase in the amount of dust, reaching 75.4 MPa for 20% CKD, which means an 18.8% decrease in strength. After 28 days of maturation, the control sample reached a value of 110.3 MPa, and an increase to 125.5 MPa and 125.2 MPa for 5% and 10% dust, respectively. The addition of 15% and 20% dust resulted in a decrease of 5.1% and 8.4%, respectively. The 0% CKD sample reached 125.6 MPa after 730 days of maturation. After adding 5%, 10%, and 15% CKD, these values increased to 137.9 MPa, 152 MPa and 138.3 MPa (increases of 9.8%, 21%, and 10.1%), respectively. Only with the addition of 20% CKD was there a slight decrease of 0.7%. As shown by the results in the case of HPC, the addition of dust from the cement kiln had a negative impact on the compressive strength only in the case of 7 days of curing. An increase was observed for 28 days up to 10% CKD and a slight decrease when the addition was increased to 20%. In the case of analysis after 2 years of concrete maturation, the strength increases up to 15% CKD, until it reaches a value lower by only 0.7% with 20% CKD.

A reduction in the compressive strength of hardened concrete mixtures with a high content of CKD arises from an increase in the content of alkalis, sulfates, and chlorides, and a reduction in the value of slag and clinker. This affects the amount of C–S–H and aluminate hydrates, which leads to the formation of significant amounts of hydrated sulfoaluminates and chloroaluminates, causing the expansion and softening of hardened concrete [35].

**Figure 6:**
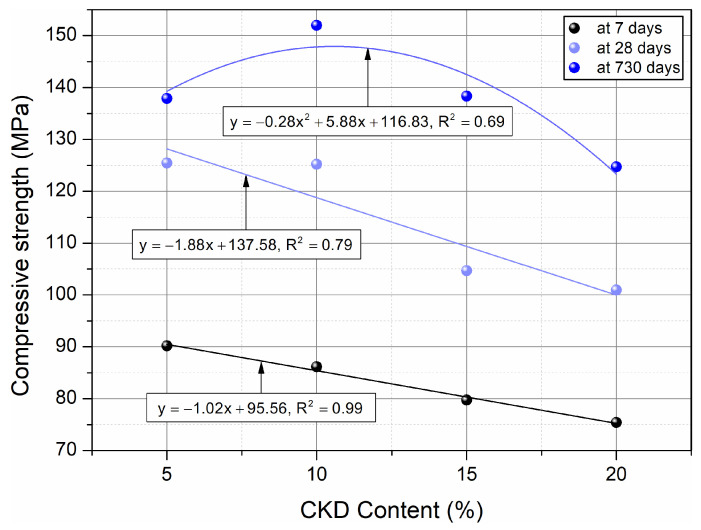
Influence of age on compressive strength of HPC and correlations between compressive strength and amount of CKD.

Figure 6 demonstrates the linear relationships (for 7 and 28 days of curing) and the quadratic relationship (for 730 days of curing) between the compressive strength of HPC and the variable percentage addition of CKD after 7, 28, and 730 days of hardening of the samples. Linear correlations showed a linear deterioration of the compressive strength of HPC samples with increasing dust addition from 5% to 20%, and the quadratic correlation showed an initial improvement (for 10–15% CKD) and then a deterioration of strength at 20%. The predicted compressive strength equations for HPC samples with CKD of different ages are shown in Figure 6. It can be seen that only after 730 days of aging, the HPC samples with CKD addition did not show a strong R^2^, reaching 0.69, i.e., slightly below the acceptable value of 0.7.

Based on the results of the compressive strength of HPC, it can be concluded that the optimal amount of CKD addition is 15%, due to a slight decrease in strength (5.1%) after 28 days of maturation and an increase after 730 days (10.1%). At the same time, there was a rather significant 13.4% decrease in compressive strength after 7 days, which should be taken into account in future applications of the material. Increasing the CKD concentration to 20% is also possible due to slight decreases of 8.4% and 0.7% for 28 and 730 days of maturation, respectively. However, the large 18% decrease in 7-day strength should also be considered.

Maslehuddin et al. [36] analyzed the effect of replacing cement with cement dust from a kiln on the compressive strength of concrete. After curing for 28 days, the compressive strength did not change when the cement was replaced with 5% CKD. An increase to 10% CKD resulted in a decrease of 10.0% and 1.0% (for concrete with cement with a lower Al_2_O_3_ content). An increase in the CKD concentration to 15% resulted in a decrease of 14.8% and 9.3% (for concrete with cement with a lower Al_2_O_3_ content). After 90 days of curing, the compressive strength of 15% CKD concrete was decreased by 13.9% and 14.2% (for concrete with cement with a lower Al_2_O_3_ content). 

Najim et al. [32] determined compressive strength for a water/cement ratio of 0.4, cement/sand 1:3, for 0%, 10%, 20%, and 30% CKD after 3, 7, and 28 days of concrete curing.

At each timepoint, a decrease in strength was measured as the amount of dust increased. The addition of 10% CKD resulted in decreases of 7, 15, and 6% for 3, 7, and 28 days. The largest decreases of up to 38% were obtained when 30% CKD was added. It was noted that the reason for the decrease in strength was an increase in the CaO content of quicklime in CKD, which increases the amount of Ca(OH)_2_ due to reaction with water. This increases internal stresses because calcium hydroxide has a larger volume than water, which weakens the hardening of the cement matrix. Moreover, the increase in porosity contributes to a decrease in maximum compressive strength. Compressive strength increases with age, which can be attributed to the development of the hydration process. Shoaib et al. [37] examined the effect of replacing Portland cement with CKD at 10%, 20%, 30%, and 40%. The mixtures were made with a w/c coefficient = 0.5. Compressive strength was determined for 1, 3, and 6 months after the samples were manufactured. The strength of the control mixture without CKD was 27 MPa, 28.5 MPa, and 43 MPa according to the maturation time. A decrease in compressive strength was observed depending on the amount of CKD added and the age of the concrete. For 10% CKD it was a decrease of 15%, 3.5%, and 1.6% after 1, 3, and 6 months, respectively. The strength loss in the case of a 40% replacement of cement with CKD was approximately 44% regardless of the age of the sample. Al-Harthy et al. [38] determined the compressive strength of concrete mixtures with the addition of CKD after 3, 7, and 28 days of curing. Mixtures containing 0, 5, 10, 15, 20, 25, and 30% CKD were prepared. The greatest decreases in compressive strength were found for the ratio w/c = 0.7 compared to w/c = 0.5. After 28 days of maturation, the samples obtained strengths of 34.4, 37.5, and 55 MPa for w/c = 0.7, 0.6, 0.5, respectively. The addition of 5% CKD in the case of maturation of 28 days had no noticeable adverse effect on the compressive strength, especially for w/c = 0.5—a decrease in strength by 1.8 and 4.5% for 10% CKD. At w/c = 0.6 the decrease was 12.4 and 18.0% for 5 and 10% CKD, respectively. At w/c = 0.7 the decrease was 8.0 and 13.0% for 5 and 10% CKD. At 30% CKD, the compressive strength decreased by 31%, 29%, and 22% for w/c 0.7, 0.6, and 0.5, respectively. Based on the results, it can be concluded that as the amount of water increases, the addition of CKD reduces the compressive strength of conventional concrete to a greater extent. A larger amount of water requires a larger amount of cement to bind all the ingredients of the mixture, which turns out to be insufficient when replacing cement with dust from a cement kiln.

To summarize, the replacement of cement with CKD up to 10% in HPC improves its compressive strength (max. to 20%) by utilizing the pozzolanic effect, accelerating the production of C–S–H gel, and reducing the quantity of CH. The pozzolanic effect in HPC causing the strength improvement is significantly influenced by the high percentage of SiO_2_ in the silica fume, rather than in the CKD (where SiO_2_ does not exceed 8%). However, as the amount of replacement CKD increased (from 15%) above the amount of silica fume, the compressive strength of HPC began to decrease. This is due to the replacement of cement clinker, which is responsible for strength development. In addition, increasing amounts of chloride present in HPC cause the crystallization of hydration products, which results in the opening of the pore system leading to a decrease in strength. The chloride ions of CKD are also involved in chemical reactions yielding chloro-aluminate hydrate, which causes softening of the material [1].

### 3.4. Splitting Tensile Strength

**Figure 7:**
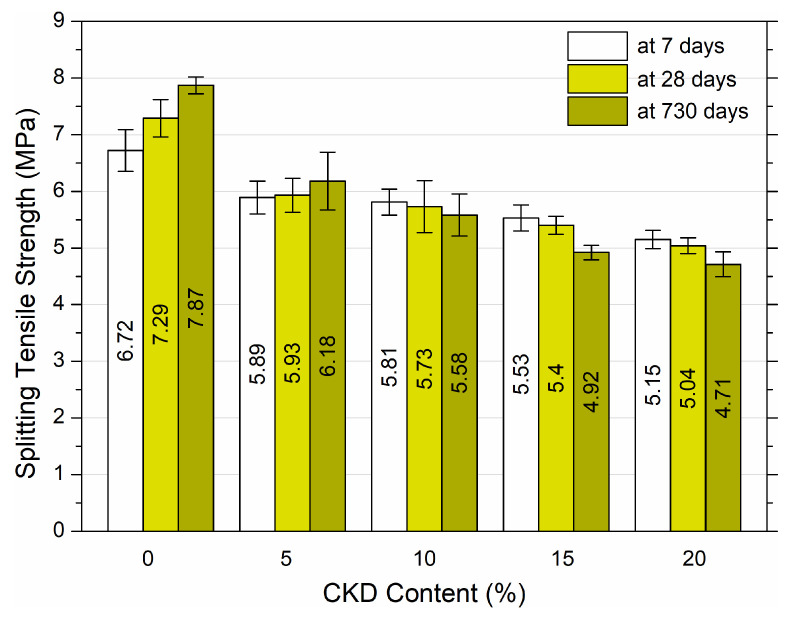
Splitting tensile strength of HPC at 7, 28, and 730 days of maturation with variable additive of CKD.

**Figure 8:**
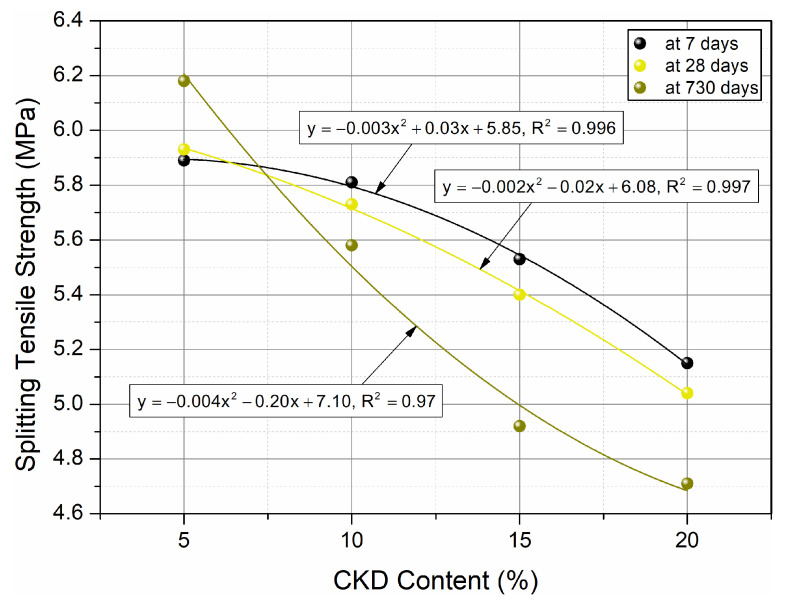
Impact of age on splitting tensile strength of HPC and correlations between splitting tensile strength and amount of CKD.

Splitting tensile strength tests were performed for the prepared cubic samples. The results after 7, 28, and 730 days of curing are shown in Figure 7. Graphs of quadratic functions with a very strong fit of the models to the results of splitting tensile strength tests and formulas for individual curing periods are illustrated in Figure 8.

The data presented in Figure 7 show the average splitting tensile strength values of three samples along with the standard deviation for each mixture. Based on the results, it was found that as the amount of CKD increased, the splitting tensile strength decreased. The strength after 7 days of maturation reached the value of 6.72 MPa for 0% CKD and 5.15 MPa for 20% CKD—a decrease of 23.36%. For samples after 28 days of maturation, there was a proportional decrease in strength, depending on the amount of CKD added. The control sample achieved 7.29 MPa, while the remaining cubes demonstrated decreases in splitting tensile strengths of 18.7%, 21.4%, 25.9%, and 30.9% for 5%, 10%, 15% and 20% CKD, respectively. The greatest decrease in strength occurred in samples after 730 days of maturation. The 0% CKD sample reached 7.87 MPa after 730 days and the addition of dust resulted in decreases of 21.5%, 29.1%, 37.5% and 40.2% in samples containing 5%, 10%, 15%, and 20% CKD, respectively. The addition of CKD had a significant decreasing effect on the splitting tensile strength value. Even the small amount of 5% CKD resulted in reductions of 18.7% and 21.5% at 28 and 730 days of maturation. These results indicate that the use of CKD additives in concrete in situations where it is necessary to obtain high tensile strength should be particularly analyzed.

The impact of CKD after 1, 3, and 6 months of maturation was analyzed in [37]. Cement was replaced with dust in amounts of 10%, 20%, 30%, and 40%. The mixtures were prepared with a w/c ratio of 0.5. It was observed that the splitting tensile strength decreased with the increase in dust concentration. For 10% CKD, a decrease of 13%, 12%, and 13% was found for 1, 3, and 6 months of maturation, respectively. The greatest decrease occurred for the addition of 40% dust. The strength decreased by 39%, 41%, and 41% for 1, 3, and 6 months after molding, respectively. A decrease in splitting strength with concrete age was also observed. Al-Harthy et al. [38] examined the effect of replacing part of the cement in the mixture with CKD. Samples with the addition of 5%, 10%, 15%, 20%, 25%, and 30% of dust were manufactured, with w/c ratio of 0.5, 0.6, and 0.7 and the tensile strength of the samples was analyzed after 3, 7, and 28 days. The tensile strength of the control sample (0% CKD) after 28 days reached 3.8 MPa, 3.85 MPa, and 4.7 MPa for w/c = 0.7, 0.6, and 0.5, respectively. The greatest decrease in strength occurred at w/c = 0.7 and amounted to 23.7% for 5%, 30.3% for 10% CKD and a maximum decrease of 36.8% for 20% CKD. For the w/c ratio = 0.6, the strength decreased by 3.9% for 5% and 10% and by a maximum of 27.3% for 25% CKD. In the case of w/c = 0.5 for the addition of 5% and 10% CKD, there was an increase in strength of 6.5% and a notable decrease of 21.3%. In another study, the tensile strength of concrete after 7, 28, and 90 days of maturation increased with the increase in dust content and the age of the samples [31]. The control sample (0% CKD) reached 6.4 MPa and 7.2 MPa at 28 and 90 days post molding. The greater the dust addition, the more the strength increased, attaining 7.45 MPa and 8.15 MPa at 28 and 90 days, respectively.

In summary, the addition of CKD to HPC decreased the splitting tensile strength and showed a gradual decrease as the amount of replacement CKD increased. This was attributed to the increasing percentage of CKD, which does not provide a good bond between the aggregate and the cement paste. Thus, HPC provides a weaker bond between the aggregate particles, which lowers the splitting tensile strength.

The quadratic correlations between splitting tensile strength and CKD volume content for high performance concrete for curing ages of 7, 28, and 730 days, respectively, with almost ideal R^2^ values, are shown in Figure 8. Splitting tensile strength of HPC with the addition of CKD at different periods of maturation can be predicted using equations describing quadratic functions.

### 3.5. Modulus of Elasiticity

**Figure 9:**
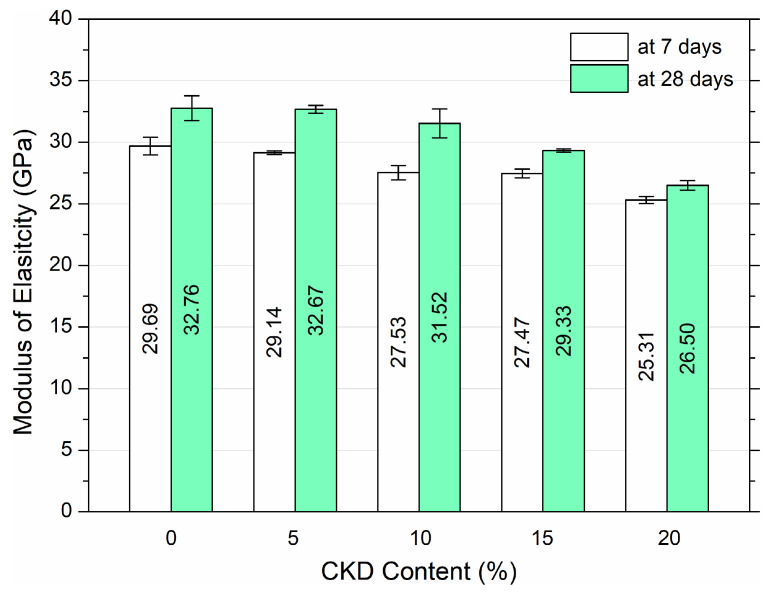
Elastic modulus of HPC at 7 and 28 days of curing with variable additive of CKD.

**Figure 10:**
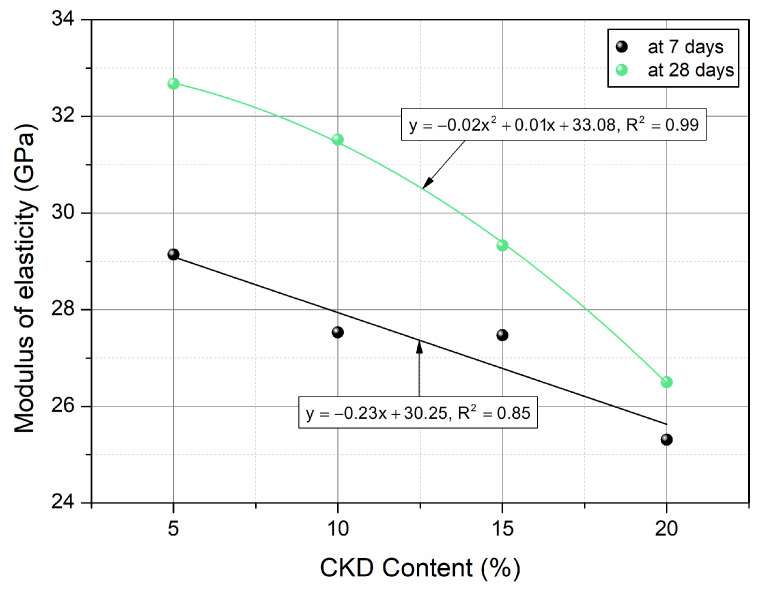
Effect of age on elastic modulus of HPC and relationships between elastic modulus and amount of CKD.

The elastic modulus of HPC was examined for cylindrical samples. The results after 7 and 28 days of maturation are presented in Figure 9. In turn, Figure 10 shows linear and quadratic models adjusted by regression to the results of tests of the elastic modulus function of HPCs with a variable addition of CKD determined at different ages of maturation.

Figure 9 displays the values of the average elastic modulus along with the standard deviation for three cylindrical samples for each mixture. Based on the results, it was found that the elastic modulus of HPC decreased with the increase in the amount of CKD. The control sample, after 7 days of maturation, reached an elastic modulus of 29.69 GPa. With the addition of CKD in the amount of 5%, 10%, 15%, and 20%, the elastic modulus decreased to 29.14 GPa, 27.53 GPa, 27.47 GPa, and 25.31 GPa, respectively, reductions of 1.9%, 7.3%, 7.5%, and 14.8%, respectively. For HPC, after 28 days of curing, there was also a decrease in the modulus proportional to the addition of CKD. The control sample value was 32.8 GPa. With the addition of CKD at 5%, 10%, 15%, and 20%, there was a decrease to 32.67 GPa, 31.52 GPa, 29.33 GPa, and 26.5 GPa, respectively, which is a reduction of 0.3%, 3.8%, 10.5%, and 19.1%, respectively. The results of the elastic modulus for HPC with the addition of CKD indicate that the optimal replacement of cement with CKD is at the level of 15%, due to the decreases of 7.5% and 10.5% after 7 and 28 days of curing.

Linear and quadratic correlations between the modulus of elasticity and the percentage of cement dust after 7 and 28 days of concrete curing were revealed by strong R^2^ values. The modulus of elasticity of HPC with the addition of CKD after various hardening times were estimated using the equations presented in Figure 10.

Abd El-Hameed et al. [17] investigated the effect of CKD on the elastic modulus of concrete after 7, 28, 56, and 91 days. They used mixtures with the addition of 10%, 20%, 30%, and 40% CKD, for a w/c ratio of 0.45. A decrease in the elastic modulus was observed with the addition of CKD. The addition of 10% and 20% CKD resulted in no noticeable difference compared to the control mixture without CKD in all tested concrete curing periods. The addition of 30% and 40% CKD resulted in a decrease in the elastic modulus. After 7 days of concrete curing, it was a decrease of 22.7% and 42.4%, respectively, and after 91 days, there was a decrease of 41.5% and 29.3%. The effect of the addition of CKD was also determined in [39], for concrete after 7 and 28 days of curing. The value of the elastic modulus decreased with increasing amounts of CKD. There were decreases of 8%, 17%, and 25% for 10%, 20%, and 30% CKD, respectively. Replacing cement with CKD in the amount of 50% resulted in a reduction of the elastic modulus by 41%.

In conclusion, the static modulus of elasticity for all HPC ages after 7 and 28 days decreased due to an increase in the percentage of fine CKD particles. This can be attributed to the spherical shape of the CKD, which led to an increase in the air content of the HPC samples.

**Figure 11:**
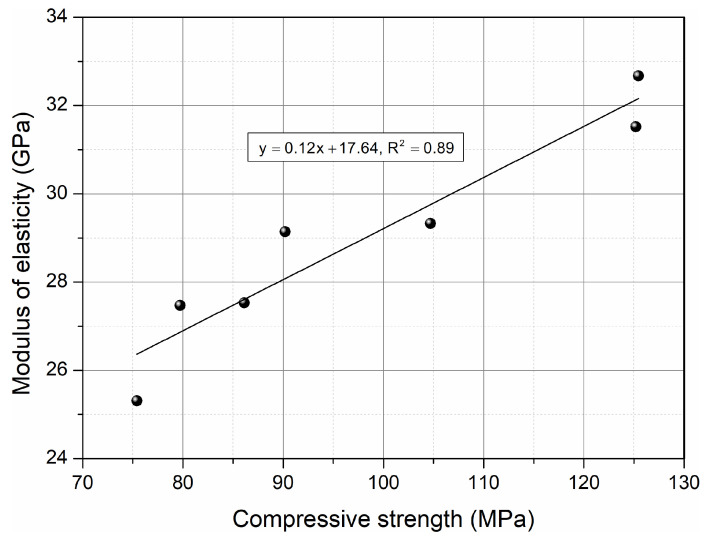
Relationship between the elastic modulus and compressive strength for HPC with added CKD.

Figure 11 shows the mutual linear relationships between the modulus of elasticity and the compressive strength of HPC with the addition of CKD in the range of 5–20%. It can be seen that as the compressive strength increased, the elastic modulus increased proportionally. The strong R^2^ value indicates that the modulus of elasticity of HPC that includes cement dust can be estimated from the compressive strength using the presented equation.

### 3.6. Ultrasonic Pulse Velocity

**Figure 12:**
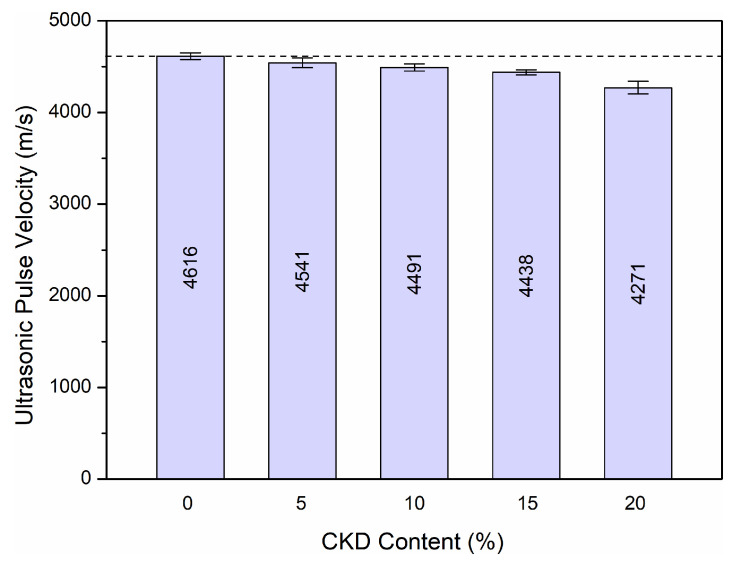
Ultrasonic pulse velocity of HPC after 730 days of maturation depending on the amount of CKD used.

The data illustrated in Figure 12 refer to the average value of the ultrasonic pulse velocity along with the standard deviation for at least ten measurements made on cylindrical samples for each mixture after 730 days of maturation.

A decrease in ultrasonic pulse velocity was found with increasing concentrations of CKD. The control sample achieved a result of 4616 m/s. Concrete with CKD 5%, 10%, 15%, and 20% obtained results of 4541 m/s, 4491 m/s, 4438 m/s, and 4271 m/s, which is a decrease of 1.6%, 2.7%, 3.9%, and 7.5%. CKD was added to the concrete in increasing amounts, which helped to limit the movement of the ultrasonic wave. The effect of adding CKD to HPC was not significant after 2 years of curing. This proves the uniformity and homogeneity of HPC with the addition of cement dust.

Ultrasonic pulse velocity was determined in [32]. During the tests, concrete samples with the addition of 0%, 10%, 20%, and 30% CKD were tested after 28 days of curing and the results obtained were 4167 m/s, 4103 m/s, 3819 m/s, and 3762 m/s, respectively. Compared to the reference sample, there was a decrease in ultrasonic pulse velocity of 1.5%, 8.4%, and 9.7%. Hussian [40] presented the results of ultrasonic pulse velocity tests for four different concrete mixtures: a control mixture with 100% cement and three mixtures with cement partially replaced by CKD and silica fume in amounts of 12.5%, 17.5%, and 22.5%. After 28 days of maturation, the tested samples achieved ultrasonic pulse velocity results of 4008 m/s, 4102 m/s, 4055 m/s, and 3864 m/s, respectively. The test results proved that 50% replacement of cement with CKD and silica fume caused a significant decrease in the homogeneity of concrete.

### 3.7. Microstructure

**Figure 13:**
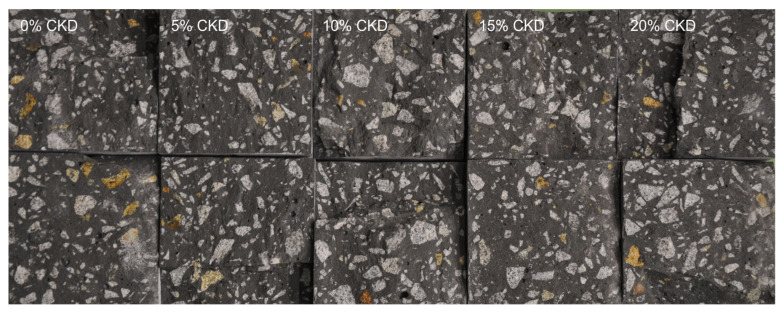
Cross-sections of HPCs cubes with variable CKD content after splitting.

The microstructure of HPC samples without the addition of CKD and with a maximum dust content of 20% was analyzed. Despite the decrease in water absorption and the increase in density with the increase in the amount of CKD, no clear differences in the split cross-sections of the cube samples were noted in Figure 13. Based on macroscopic observations, it was found that the number and size of pores, color, and arrangement of ingredients were very similar, regardless of the amount of cement dust.

**Figure 14:**
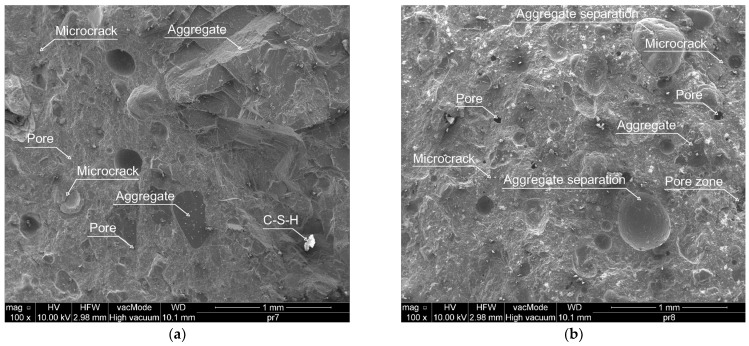

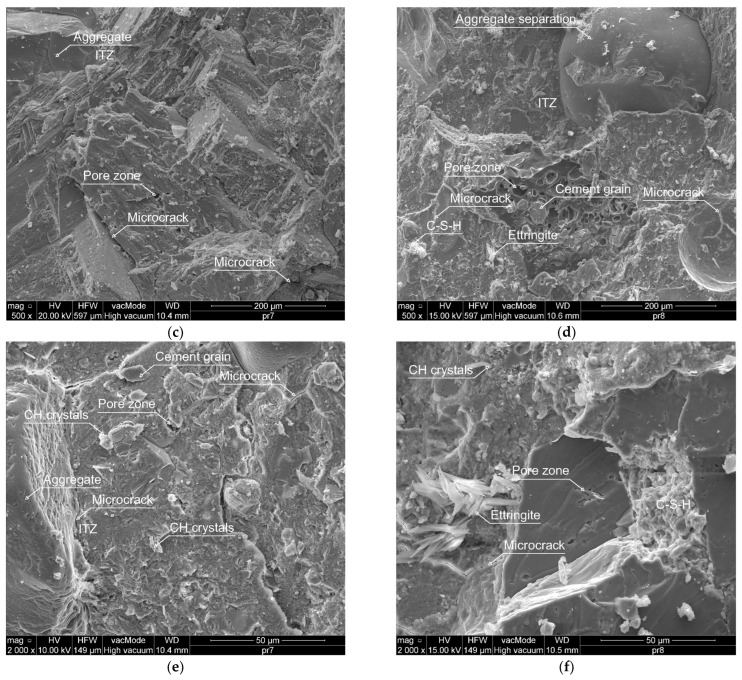
Microstructure of HPC in magnifications: 100× (**a**) 0% CKD, (**b**) 20% CKD; 500× (**c**) 0% CKD, (**d**) 20% CKD; and 2000× (**e**) 0% CKD (**f**) 20% CKD.

Using a scanning electron microscope (SEM), microphotographs were taken, based on which a qualitative and quantitative analysis of the local microstructure of HPC was carried out. Figure 14a–f shows the images of the HPC mixtures for the 0% CKD control sample and the 20% CKD sample at magnifications of 100, 500, and 2000 times. Pores and areas of their concentration, single grains of fine aggregate, microcracks, loose cement particles, CH (calcium hydroxide/portlandite) crystals, C–S–H (calcium silicate hydrate) gel, as well as locations of interfacial transition zone (ITZ) can be observed.

Based on the analysis of representative microphotographs, the HPC structure without the addition of CKD demonstrates a greater uniformity. Larger amounts of very fine pores and pore zones were observed in samples with a maximum of 20% CKD. A smaller number of pores, but with much larger dimensions, can be observed in the reference samples without the addition of CKD (see Figure 14a). The analysis of HPC microphotographs with 20% CKD also identified the occurrence of ettringite needles, which were not visible in the control sample. The heterogeneity of the concrete matrix in sample containing 20% CKD may be caused by the lower workability of this mixture, resulting in a reduction in the amount of C–S–H gel formed and its uneven distribution. C–S–H gel, which constitutes 60–70% of the total volume of the concrete mix, is a key component of cementitious materials affecting mechanical properties and durability [41]. In addition, it has been established that the microstructure of C–S–H directly improves not only the strength, but also the creep of the cement paste and shrinkage [42,43,44].

**Figure 15:**
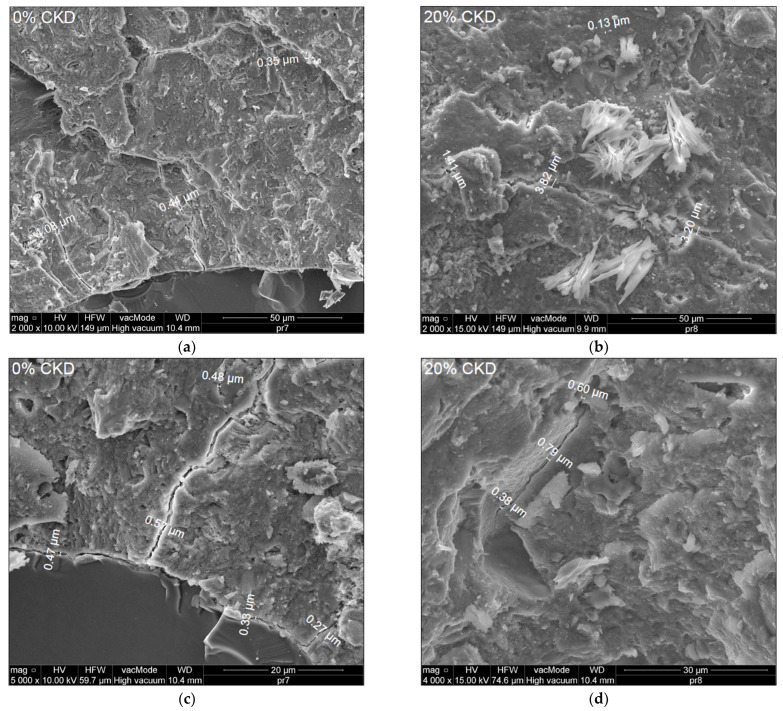
Microstructure of HPC in magnification: 2000× (**a**) 0% CKD (**b**) 20% CKD; 5000× (**c**) 0% CKD, 4000× (**d**) 20% CKD.

Figure 15a–d shows microphotographs of 0 and 20% CKD mixtures at magnifications of 2000× (a,b) and 5000× and 4000× (c,d), taken to quantify the occurrence of microcracks.

Kunal et al. [33] investigated the effect of the addition of 5, 10, and 15% CKD on the properties of concrete. Based on microscopic photos, they demonstrated the influence of CKD on the microstructure of concrete. An increase in the formation and deposition of calcium silicate hydrate gel and the occurrence of ettringite needles was found with the addition of CKD. Peethamparan et al. [11] also found that samples with the addition of CKD contained significant amounts of calcium hydroxide and ettringite needles.

Analysis of microphotographs determined that the dimensions of the microcracks were in the range of 0.27–1.08 µm for 0% CKD and 0.13–3.82 µm for 20% CKD. A clear increase in the width of microcracks was found for HPC containing 20% CKD (see Figure 15) due to greater heterogeneity of the microstructure, as observed in Figure 14.

**Figure 16:**
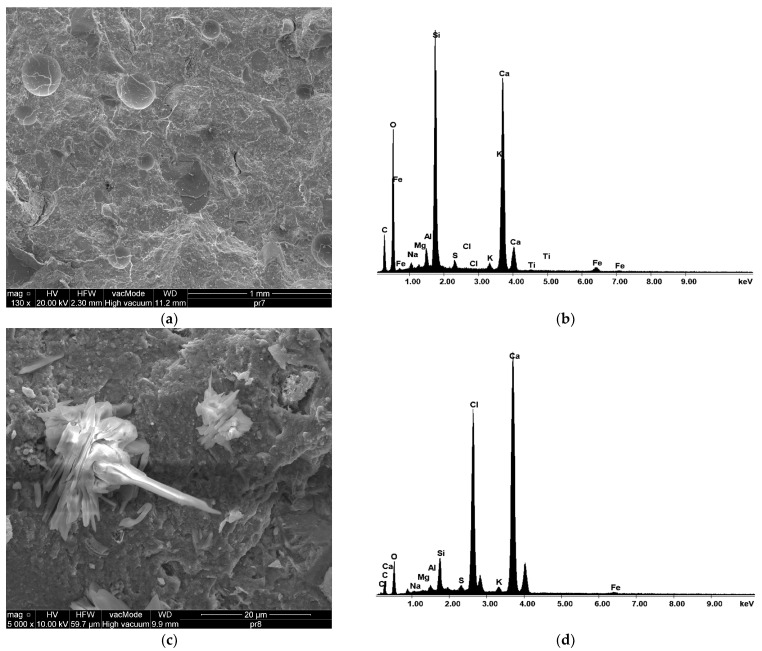
Microphotographs and EDX analysis of HPC structure: (**a**,**b**) 0% CKD; (**c**,**d**) 20% CKD.

The results of the EDX microanalysis are shown in Figure 16 and Table 3. These findings show that the interaction of clinker phases saturated with 20% cement dust was less intense compared to the reference mixture. C–S–H will be formed when portlandite reacts with silica fume [45,46,47]. The high alkali content in CKD significantly increases the solubility of the clinker phases [48].

The EDX results for HPC illustrate the effect of the addition of 20% CKD on the microstructure, relative to the control mixture. The low presence of C–S–H observed in the HPC samples with cement dust may be responsible for the effects. This feature is also a key contributing factor to the formation of internal microcracks and numerous pore clusters in the HPC containing 20% CKD. The value of the Ca/Si ratio presented in Table 3 reflects the favorable pozzolanic reaction rate. These results confirm the active role of silica fume in strengthening the microstructure of HPC without the addition of CKD [49]. Based on these results, it can be seen that the percentage of calcium in the HPC mixture with 20% CKD is higher than in the reference mixture, which indicates a quicker setting time in HPC with cement dust.

## 4. Conclusions

This research studied the impact of using variable concentrations of cement kiln dust (CKD) on the mechanical and microstructure characteristics of high-performance concrete (HPC) at various timepoints. The properties of HPC with CKD at concentrations of 5%, 10%, 15%, and 20%, with a w/c ratio of 0.25, and after aging for 7, 28, and 730 days were tested in relation to the reference HPC. Based on the results, it can be concluded that:

- The addition of CKD had a positive effect on the absorbability of HPC. As the amount of CKD increased, the water demand was reduced and reached a minimum value of 2.21% when the concentration of CKD was 20%.
- As the amount of CKD increased, the porosity of the HPC decreased and the bulk density increased. Porosity reached a minimum value of 5% when the concentration of CKD was 20%.
- The compressive strength of HPC increased when the concentration of CKD was 10% after 28 and 730 days of maturation. When the concentration of CKD was 15%, the strength of the HPC was slightly higher than that of the reference sample, and when the concentration of CKD was 20%, it was slightly lower.
- The value of tensile splitting strength decreased proportionally as the amount of CKD increased. Even the addition of 5% CKD caused a decrease of approximately 20% after 28 and 730 days of maturation. The maximum tensile splitting strength decreases were 30.9% and 40.2% when the concentration of CKD was 20% after 28 and 730 days of curing, respectively.
- The elastic modulus gradually decreased with the amount of CKD added. In the case of 10% CKD, after 28 days of maturation, the HPC modulus decreased by only 3.8%. The maximum decline attained at a CKD concentration of 20% was 19.1%.
- The ultrasonic pulse velocity decreased as the concentration of CKD increased. This was caused by a reduction in the homogeneity of the HPC matrix. The largest decrease of 7.5% occurred when the concentration of CKD was 20%.
- SEM and EDX analyses showed a greater heterogeneity of the microstructure and a decrease in the presence of C–S–H with increasing CKD, resulting in the formation of wider microcracks and ettringite needles.
- The Ca/Si value is a useful indicator for evaluating the pozzolanic reaction. CKD actively deteriorated the microstructure of HPC, increasing the solubility of the clinker phases and reducing the amount of C–S–H gel formed.

The research has revealed that CKD can be a partial substitute for cement in the production of high-performance and even ultra-high-performance concrete. Rational addition of CKD at concentrations of up to 15%, while maintaining the optimal w/c ratio, type of aggregate, and cement type, does not negatively affect all HPC parameters. The lack of a noticeable difference or a slight deterioration of some characteristics does not disqualify the partial replacement of cement with CKD. At the same time, the benefits of reducing the cost of the HPC mixture and the use of waste dust, which may have a negative impact on the environment, should be taken into account.

## Figures and Tables

**Table 1:** Basic composition of high-performance concrete for 1 m^3^ (without CKD additive).

Ingredient	Quantity
Cement [kg]	748
Sand [kg]	435
Coarse aggregate [kg]	870
Silica fume [kg]	187
Water [L]	230
Superplasticizer [L]	20

**Table 2:** Chemical composition of CKD.

Compound	Content [% by Weight]
CaO	35.50
K_2_O	18.00
Cl	15.28
SiO_2_	7.41
SO_3_	4.73
Fe_2_O_3_	2.63
Al_2_O_3_	1.93
Na_2_O	1.36
MgO	0.48
Others	0.09

**Table 3:** EDX analysis results of HPC.

Formula	Atom [%] 0% CKD	Atom [%] 20% CKD
Calcium	12.11	17.02
Carbon	24.24	30.87
Silicon	12.94	3.27
Iron	0.62	0.18
Aluminum	1.14	0.97
Oxygen	47.20	34.20
Sodium	0.61	0.85
Magnesium	0.21	0.58
Sulfur	0.47	0.54
Chlorine	0.05	11.07
Potassium	0.35	0.44
Titanium	0.07	—
Ca/Si ratio	0.94	5.20

## Data Availability

The data that support the findings of this study are available from the corresponding authors, P.S. and K.B., upon reasonable request.
